# Supplementary material for: MetaRibo-Seq measures translation in microbiomes
Source: Nat Commun. 2020 Jun 29;11:3268. doi: 10.1038/s41467-020-17081-z (PMC7324362; doi:10.1038/s41467-020-17081-z)
Supplement: Supplementary file 10 — Supplementary Data 7 [file 41467_2020_17081_MOESM10_ESM.zip › File2/Confidence_High_Taxonomy/286428_out.krona.html]

Javascript must be enabled to view this page.

members
magnitude
magnitudeUnassigned
count
unassigned
taxon
rank

286428\_out

238

2
5
superkingdom
238

SRS015369\_contig\_number\_contig-100\_1164.53440SRS049959\_contig\_number\_24110SRS063040\_contig\_number\_22020SRS064557\_contig\_number\_1987SRS1041145\_contig\_number\_contig-100\_4426.194475

1239
4
phylum

class
4
186801

186802
4
order

2
541000
family

genus
216851
1

1
853

SRS148319\_contig\_number\_16709
species

genus
1
1263

species
1
41978

SRS016954\_contig\_number\_4041

2
186803
family

species

SRS019068\_contig\_number\_18762SRS143991\_contig\_number\_18053
39491
2

976
229
phylum

200643
229
class

229
171549
order

family
3
171552

838
2
genus


SRS078419\_contig\_number\_24644
165179
1
species

1
59823

SRS048164\_contig\_number\_15308
species

genus
1
577309

1
454154

SRS063370\_contig\_number\_9782
species

171550
1
family

genus
239759
1

species
1
328814

SRS019445\_contig\_number\_26625

family
815
225

225

SRS011302\_contig\_number\_contig-100\_3135.166680SRS012969\_contig\_number\_25707SRS013158\_contig\_number\_contig-100\_2456.38673SRS013521\_contig\_number\_3243SRS014923\_contig\_number\_contig-100\_2238.213571SRS015694\_contig\_number\_contig-100\_16865.96149SRS015794\_contig\_number\_4931SRS017103\_contig\_number\_32766SRS017433\_contig\_number\_contig-100\_2641.96979SRS018351\_contig\_number\_contig-100\_2898.93573SRS019397\_contig\_number\_contig-100\_1430.81854SRS019496\_contig\_number\_16506SRS024132\_contig\_number\_52117SRS024331\_contig\_number\_contig-100\_4063.207673SRS043841\_contig\_number\_contig-100\_16302.16303SRS049896\_contig\_number\_26555SRS049995\_contig\_number\_contig-100\_4987.198335SRS050974\_contig\_number\_contig-100\_7290.7291SRS052027\_contig\_number\_contig-100\_4510.109733SRS052697\_contig\_number\_38929SRS077127\_contig\_number\_contig-100\_690.119558SRS077849\_contig\_number\_26942SRS078242\_contig\_number\_26677SRS078665\_contig\_number\_contig-100\_5232.68756SRS098644\_contig\_number\_contig-100\_12803.206477SRS103987\_contig\_number\_contig-100\_10311.163995SRS1041139\_contig\_number\_7SRS1041157\_contig\_number\_contig-100\_4451.50456SRS143372\_contig\_number\_contig-100\_6403.79283SRS146812\_contig\_number\_contig-100\_7589.283835SRS147346\_contig\_number\_contig-100\_1292.172635SRS893187\_contig\_number\_17986SRS893341\_contig\_number\_contig-100\_3764.96890SRS893366\_contig\_number\_18207
816
34
genus

species
28116

SRS016840\_contig\_number\_1592SRS077194\_contig\_number\_20883
2


SRS1041138\_contig\_number\_10211
2292944
1
species

817

SRS063489\_contig\_number\_647
1
species

species

SRS075984\_contig\_number\_22799SRS145497\_contig\_number\_20458
1263054
2

species
1
2292921

SRS143523\_contig\_number\_20763

1
1263038

SRS053649\_contig\_number\_18280
species

46506

SRS017859\_contig\_number\_1942SRS019161\_contig\_number\_7218SRS022137\_contig\_number\_10821SRS1041137\_contig\_number\_17406SRS140492\_contig\_number\_11898SRS149181\_contig\_number\_12081
6
species

species
25
1946089

SRS011084\_contig\_number\_19060SRS015890\_contig\_number\_15828SRS016095\_contig\_number\_14829SRS017307\_contig\_number\_12633SRS018133\_contig\_number\_13070SRS018427\_contig\_number\_28251SRS023526\_contig\_number\_13936SRS023971\_contig\_number\_166SRS045826\_contig\_number\_19960SRS054905\_contig\_number\_17075SRS062427\_contig\_number\_10413SRS064232\_contig\_number\_2176SRS077294\_contig\_number\_17813SRS077392\_contig\_number\_22358SRS1041116\_contig\_number\_8718SRS104693\_contig\_number\_4557SRS1055095\_contig\_number\_380SRS140513\_contig\_number\_8263SRS144183\_contig\_number\_28711SRS147346\_contig\_number\_9886SRS147653\_contig\_number\_770SRS148196\_contig\_number\_25170SRS893172\_contig\_number\_9130SRS893252\_contig\_number\_17747SRS893378\_contig\_number\_12565

2292927

SRS056273\_contig\_number\_32168
1
species

species

SRS011134\_contig\_number\_22714SRS011239\_contig\_number\_23254SRS011405\_contig\_number\_15186SRS013476\_contig\_number\_28022SRS013800\_contig\_number\_23817SRS013940\_contig\_number\_6431SRS013951\_contig\_number\_33754SRS014235\_contig\_number\_28751SRS014287\_contig\_number\_16360SRS014313\_contig\_number\_3984SRS014683\_contig\_number\_4016SRS014855\_contig\_number\_10822SRS014948\_contig\_number\_20304SRS014979\_contig\_number\_3716SRS015065\_contig\_number\_contig-100\_1265.219023SRS015190\_contig\_number\_4806SRS015431\_contig\_number\_35212SRS015578\_contig\_number\_30320SRS015579\_contig\_number\_14075SRS015854\_contig\_number\_16882SRS016018\_contig\_number\_3612SRS016056\_contig\_number\_6037SRS016989\_contig\_number\_7092SRS017521\_contig\_number\_50230SRS017701\_contig\_number\_10656SRS017745\_contig\_number\_2390SRS017821\_contig\_number\_29496SRS018575\_contig\_number\_7349SRS018623\_contig\_number\_7452SRS018836\_contig\_number\_3466SRS018888\_contig\_number\_4684SRS018936\_contig\_number\_1047SRS018984\_contig\_number\_14269SRS019030\_contig\_number\_12639SRS019267\_contig\_number\_contig-100\_393.61234SRS019685\_contig\_number\_27887SRS019693\_contig\_number\_1533SRS019808\_contig\_number\_19964SRS021153\_contig\_number\_4374SRS021219\_contig\_number\_contig-100\_7166.75024SRS021484\_contig\_number\_13609SRS021948\_contig\_number\_contig-100\_1949.180549SRS022524\_contig\_number\_9516SRS022609\_contig\_number\_4882SRS024009\_contig\_number\_8376SRS024435\_contig\_number\_37061SRS042628\_contig\_number\_20817SRS043411\_contig\_number\_17413SRS043768\_contig\_number\_29615SRS045244\_contig\_number\_3323SRS045713\_contig\_number\_contig-100\_2120.109504SRS046502\_contig\_number\_4839SRS046717\_contig\_number\_1348SRS048060\_contig\_number\_5124SRS048870\_contig\_number\_19194SRS049402\_contig\_number\_15113SRS050026\_contig\_number\_9782SRS050422\_contig\_number\_36001SRS050801\_contig\_number\_1718SRS050925\_contig\_number\_14552SRS050998\_contig\_number\_11718SRS053214\_contig\_number\_contig-100\_3572.124105SRS053335\_contig\_number\_7931SRS055017\_contig\_number\_31853SRS055982\_contig\_number\_11516SRS056519\_contig\_number\_10556SRS057480\_contig\_number\_3065SRS058723\_contig\_number\_6894SRS058770\_contig\_number\_23311SRS062654\_contig\_number\_27728SRS063518\_contig\_number\_30331SRS064757\_contig\_number\_14324SRS075078\_contig\_number\_24852SRS075716\_contig\_number\_3319SRS075821\_contig\_number\_630SRS075878\_contig\_number\_22763SRS075963\_contig\_number\_25SRS076804\_contig\_number\_12875SRS077231\_contig\_number\_18847SRS077335\_contig\_number\_2278SRS077502\_contig\_number\_12630SRS077589\_contig\_number\_7967SRS077730\_contig\_number\_contig-100\_3085.114546SRS078176\_contig\_number\_7769SRS098061\_contig\_number\_10370SRS098571\_contig\_number\_16571SRS098717\_contig\_number\_13416SRS101433\_contig\_number\_7307SRS104084\_contig\_number\_11215SRS1041031\_contig\_number\_21204SRS1041032\_contig\_number\_16163SRS1041039\_contig\_number\_20428SRS1041091\_contig\_number\_18495SRS1041134\_contig\_number\_565SRS1041141\_contig\_number\_10642SRS104311\_contig\_number\_18685SRS104975\_contig\_number\_2804SRS1054691\_contig\_number\_13668SRS1055022\_contig\_number\_12977SRS1055038\_contig\_number\_3460SRS1055049\_contig\_number\_11254SRS1055069\_contig\_number\_10125SRS1055083\_contig\_number\_1905SRS142503\_contig\_number\_24790SRS142542\_contig\_number\_contig-100\_1389.159458SRS142781\_contig\_number\_11521SRS142923\_contig\_number\_15045SRS142980\_contig\_number\_contig-100\_3645.3646SRS143148\_contig\_number\_contig-100\_2237.136502SRS143181\_contig\_number\_10400SRS143342\_contig\_number\_3791SRS143466\_contig\_number\_26482SRS143895\_contig\_number\_38061SRS144362\_contig\_number\_6355SRS144506\_contig\_number\_35140SRS144603\_contig\_number\_12119SRS147088\_contig\_number\_1861SRS147271\_contig\_number\_42441SRS147557\_contig\_number\_25147SRS148253\_contig\_number\_11071SRS148424\_contig\_number\_17695SRS148721\_contig\_number\_3147SRS148784\_contig\_number\_8530SRS148817\_contig\_number\_contig-100\_1404.85850SRS149075\_contig\_number\_contig-100\_2098.104234SRS149244\_contig\_number\_12766SRS149879\_contig\_number\_6534SRS892882\_contig\_number\_contig-100\_176.83330SRS893230\_contig\_number\_8839SRS893231\_contig\_number\_1432SRS893256\_contig\_number\_28SRS893270\_contig\_number\_22810SRS893279\_contig\_number\_4153SRS893292\_contig\_number\_3592SRS893300\_contig\_number\_11492SRS893358\_contig\_number\_728SRS971275\_contig\_number\_22834SRS971276\_contig\_number\_9271
820
138

species
3
818

SRS024625\_contig\_number\_410SRS065504\_contig\_number\_9382SRS1041142\_contig\_number\_1364

1
1897051

SRS014459\_contig\_number\_contig-100\_1427.232814
species

species
3

SRS062701\_contig\_number\_5938SRS097920\_contig\_number\_14568SRS893373\_contig\_number\_6231
246787


SRS076756\_contig\_number\_13435SRS1041164\_contig\_number\_5435SRS104327\_contig\_number\_1785SRS1054716\_contig\_number\_16401SRS893253\_contig\_number\_234SRS893295\_contig\_number\_9295
821
6
species
